# Central mucoepidermoid carcinoma arising directly from a glandular odontogenic cyst of the mandible: a case report

**DOI:** 10.1186/s13000-021-01124-0

**Published:** 2021-07-11

**Authors:** Satoshi Maruyama, Taisuke Mori, Manabu Yamazaki, Tatsuya Abé, Eijitsu Ryo, Hiroyuki Kano, Go Hasegawa, Jun-ichi Tanuma

**Affiliations:** 1 Oral Pathology Section, Department of Surgical Pathology, Niigata University Hospital, 1-754 Asahimachi-dori, Chuo-ku, 951-8520 Niigata, Japan; 2 Department of Pathology, Uonuma Institute of Community Medicine, Niigata University Medical and Dental Hospital, 4132 Urasa, Minami Uonuma-shi, 949-7302 Niigata, Japan; 3 Department of Diagnostic Pathology, National Cancer Center Hospital, 5-1-1 Tsukiji, Chuo-ku, 104-0045 Tokyo, Japan; 4 Division of Oral Pathology, Department of Tissue Regeneration and Reconstruction, Faculty of Dentistry, Niigata University Graduate School of Medical and Dental Sciences, 2-5274 Gakkoucho-dori, Chuo-ku, 951-8514 Niigata, Japan; 5 Department of Oral Surgery, Uonuma Institute of Community Medicine, Niigata University Medical and Dental Hospital, 4132 Urasa, Minami Uonuma-shi, 949-7302 Niigata, Japan

**Keywords:** Central mucoepidermoid carcinoma, Glandular odontogenic cyst, Immunohistochemistry, Cytokeratin 13, *MAML*-2

## Abstract

**Background:**

Central mucoepidermoid carcinoma (MEC) is a rare salivary gland tumor that affects the jawbone. Glandular odontogenic cyst (GOC) is also a rare odontogenic developmental cyst with glandular differentiation. GOC shares some histological features with central MEC, and a pre-existing GOC can develop into central MEC. Here, we present a rare case of central MEC developed directly from a pre-existing GOC of the mandible.

**Case presentation:**

A 67-year-old Japanese man presented with a cystic lesion in the right third molar region. Histologically, the biopsy specimen demonstrated both typical findings of a GOC component lined with non-keratinized squamous epithelium and a recognizable component of central MEC consisting of polycystic nests with mucous cells, intermediate cells, and epidermoid cells in the cyst wall. The results from the immunohistochemistry for cytokeratin (CK) profiling demonstrated that, while both central MEC and GOC expressed CKs 7, 14, 18, and 19, CK13 was interestingly exclusively expressed in GOC. Fluorescence in-situ hybridization (FISH) revealed the rearrangement of the *Mastermind like (MAML)*-2 gene in both the MEC and GOC components.

**Conclusions:**

Our case suggests that central MEC and GOC may be in the same spectrum of diseases caused by the rearrangement of the *MAML-2* gene. However, given that the expression profile of CK13 was completely different between central MEC and GOC, they can be considered as separate tumors. Overall, we demonstrated a rare case in which central MEC may have originated directly from the GOC.

## Background

The most common type of salivary gland tumor arising from the jaw is central mucoepidermoid carcinoma (MEC) [1]. With regard to the developmental origin, 50 % of central MECs are associated with an odontogenic cyst or unerupted tooth [1]. Glandular odontogenic cyst (GOC) is an uncommon developmental cyst and numerous histopathological features of GOC, such as eosinophilic surface cuboidal cells, intraepithelial microcysts, and mucous cells have been described [2]. GOC shares some histopathological features with central MEC, including a cystic space lined by an epithelium consisting of mucous cells and squamous cells; consequently, it may be confused with central MEC [3]. However, there is only one report of GOC transforming to central MEC [4], which identified the two components (i.e., GOC and central MEC) in the same tissue. Therefore, GOC is the most important entity in the differential diagnosis of central MEC; however, the morphological similarities make diagnosis difficult. Although immunohistochemistry for the cytokeratin (CK) profile and analysis of the *Mastermind like (MAML)*-2 gene rearrangement are reportedly useful for distinguishing GOC from central MEC, only a limited numbers of the cases have been described [3, 5–10]. The aims of this case study were to analyze the immunohistochemical expression of CKs and *MAML*-2 gene rearrangement in a case of central MEC arising directly from a GOC, and to compare the findings between GOC and central MEC.

## Case presentation

### Clinical history

**Fig. 1:**
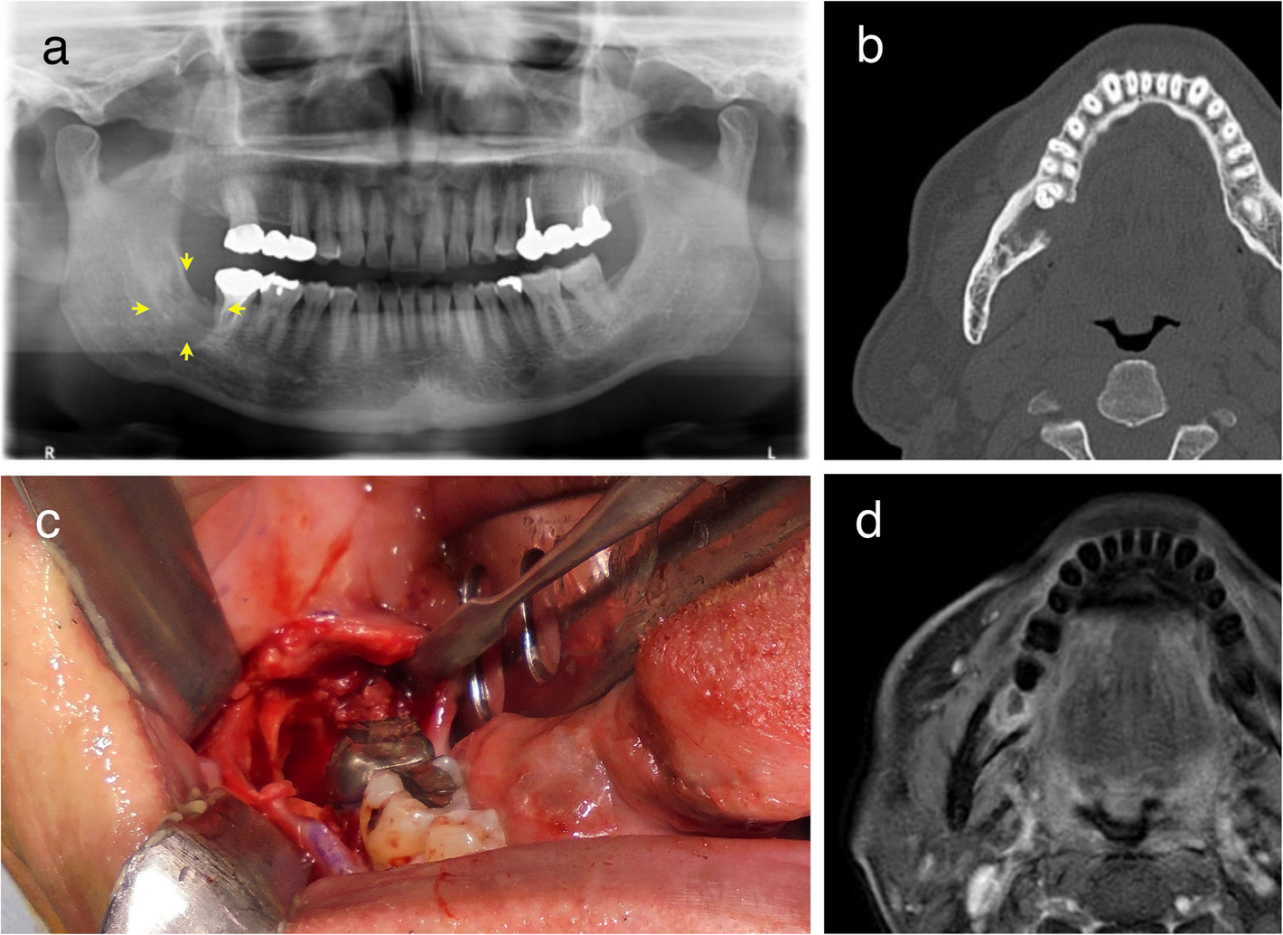
Radiological findings of the central mucoepidermoid carcinoma (MEC) arising from a glandular odontogenic cyst (GOC) of the mandible. **a** Panoramic radiograph showing a radiolucent cystic lesion (yellow arrows) in the right third molar region of the mandible. **b** CT showing a unilocular radiolucent lesion with a lingual side cortical bone resorption of the mandible. **c** Oral photography showing a cystic cavity in the same area after surgery. **d** MRI showing a contrast defect in the same area after the biopsy was taken

A 67-year-old Japanese man gave a history of being diagnosed with a cystic lesion in the right third molar region of the mandible by X-rays 11 years earlier. Subsequently, a tooth extraction had been performed. However, cyst enucleation and histopathological examination had not been carried out at that time. Eight years after the tooth extraction, he noticed a gingival swelling which lasted for three years. The medical history was only prostatic hypertrophy. On examination, a slight swelling was palpable in the gingiva of the right third molar region of the mandible. There was no fistula but a part of the bone had a defect. A panoramic radiograph revealed a radiolucent cystic lesion, measuring 10 × 12 mm in the same area (Fig. 1a, yellow arrows). A computed tomography (CT) showed an unilocular radiolucent lesion along with cortical bone resorption of the mandible on the lingual side (Fig. 1b). On the basis of clinical and radiological findings, a presumptive diagnosis of an odontogenic cyst was made and a biopsy was performed (Fig. 1c). The incisional biopsy resulted in a diagnosis of central MEC arising from a GOC as described below. Chest and abdominal CT findings were within normal limits. A magnetic resonance imaging (MRI) revealed a contrast defect in the same area (Fig. 1d). Cervical lymph node metastasis was absent on MRI. Due to the malignant nature of the tumor, as well as a history of previous surgeries, a partial mandibulectomy was performed to remove the lesion with a sufficient surgical margin and the tumor was surgically excised under general anesthesia. Following a final diagnosis of central MEC, the patient made an uneventful recovery and demonstrated no clinical evidence of recurrence in the two years following the surgery.

### Pathological findings

**Fig. 2:**
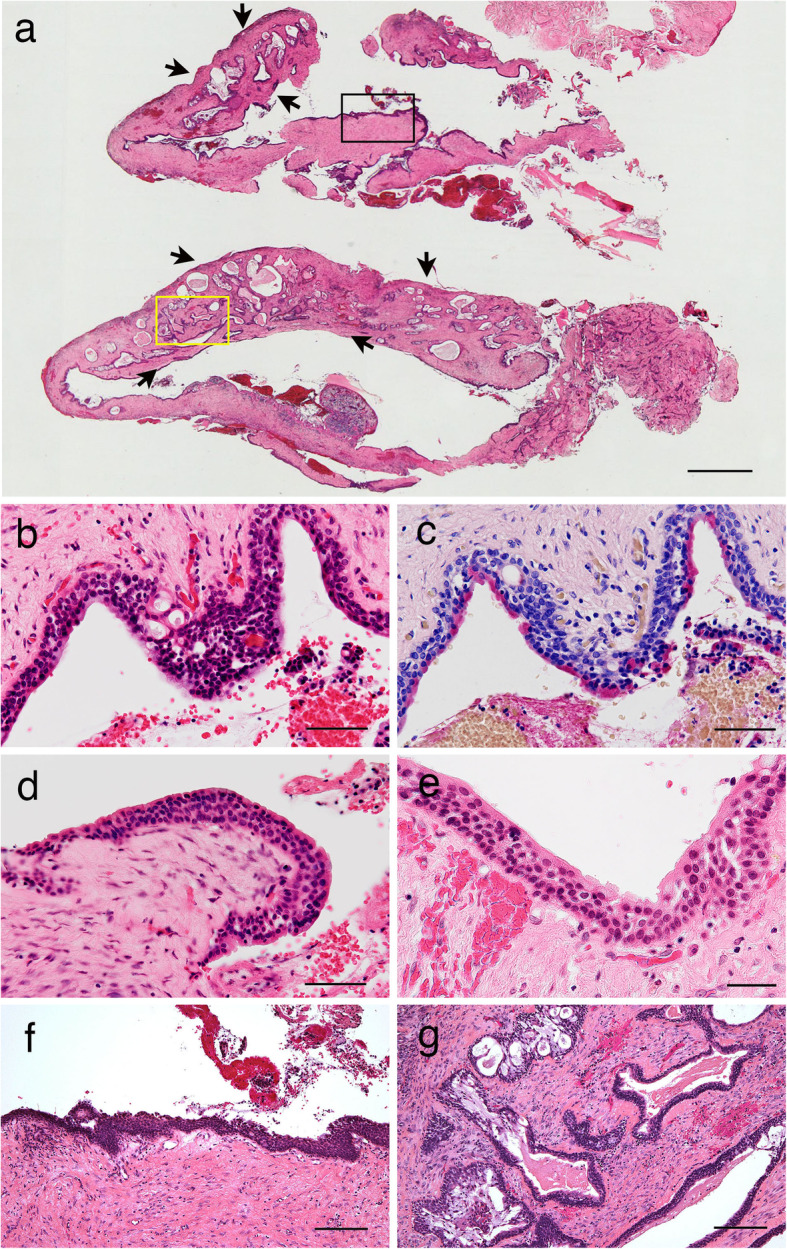
Histopathology of central MEC arising from a GOC of the mandible in a biopsy specimen. **a** The biopsy showed an enlarged unilocular cyst with thick fibrous connective tissue, along with a polycystic lesion on one side (black arrows). **b** Numerous microcysts and mucous goblet cells were observed in the cyst lining epithelium. **c** Mucous goblet cells were positive for mucicarmine staining. **d** Eosinophilic cuboidal cells were seen within the lining epithelium. **e** Ciliated cells were scattered within the non-keratinized squamous epithelial cells. **f** The cyst wall consisted of fibrous connective tissue and non-keratinizing squamous epithelium in the GOC part (black square in **a**). **g** Several cystic nests, which contained eosinophilic materials, were also found in the central MEC parts (yellow square in **a**). Hematoxylin-eosin (**a–b, d–g)**, mucicarmine stain (**c**), Scale bars, 1 mm (**a**), 20 μm (**b–e**), 100 μm (**f–g**)

Microscopic examination of the biopsy material showed an enlarged unilocular cyst (Fig. 2a). The cystic lumen was lined by epithelial cells and was surrounded by thick fibrous connective tissue. Additionally, a solid polycystic lesion was also seen on one side (Fig. 2a, black arrows). Numerous microcysts and mucous goblet cells were observed in the lining epithelium (Fig. 2b). The intraepithelial mucin in the mucous goblet cells was positive for mucicarmine staining (Fig. 2c). Eosinophilic cuboidal cells (Fig. 2d) and ciliated cells (Fig. 2e) were scattered within the non-keratinized squamous epithelial cells. These histopathological findings were suggestive of a GOC. In addition to the cyst wall consisting of fibrous connective tissue and the above-mentioned non-keratinized squamous epithelium coating the fibrous stroma (Fig. 2a, black square and 2f), the proliferation of many cystic nests containing mucous materials was observed in another part of the cyst wall (Fig. 2a, yellow square and 2 g). The lining epithelium inside several cysts consisted of a mixture of epidermoid, mucous, and intermediate cells (Fig. 2g). These findings served to confirm the diagnosis of central MEC arising from a GOC.

**Fig. 3:**
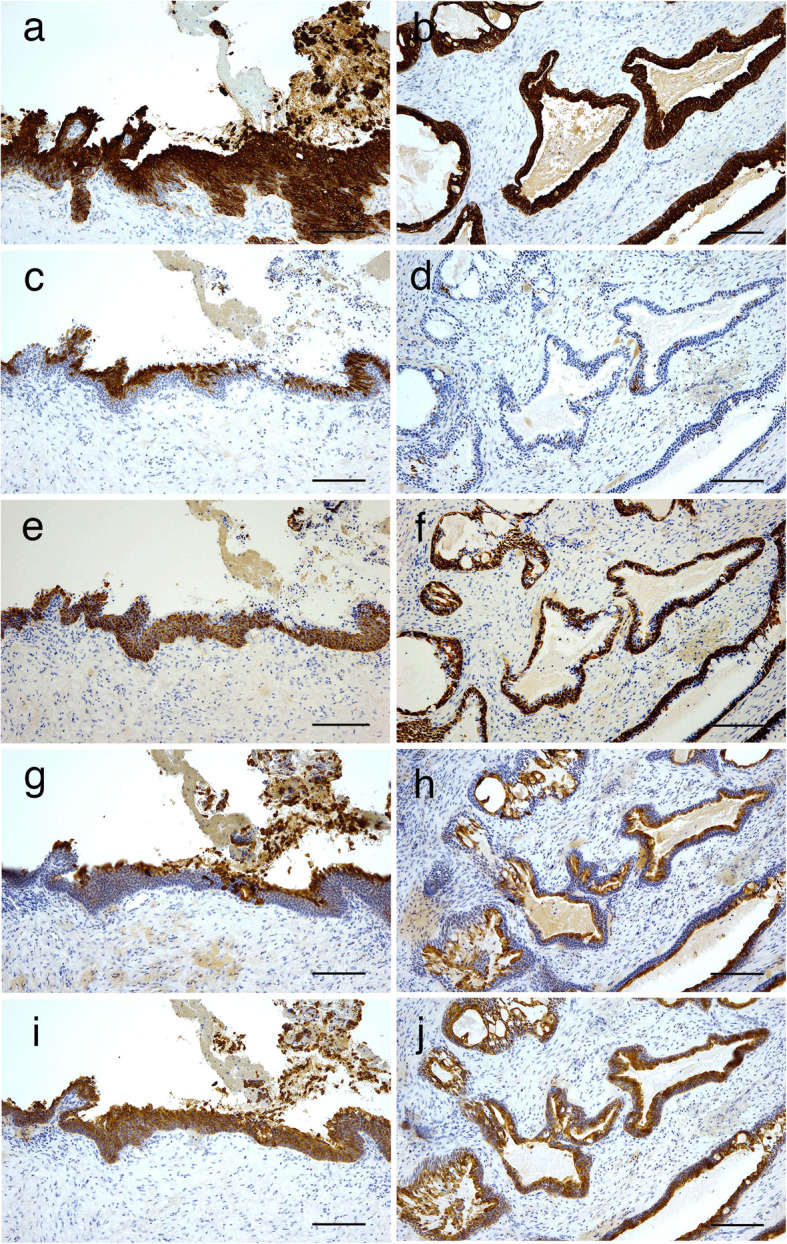
Immunohistochemical profile of keratins in central MEC arising from a GOC. Immunoperoxidase stain for (**a, b**) CK7, (**c, d**) CK13, (**e, f**) CK14, (**g, h**) CK18, and (**I, j**) CK19. GOC parts expressed CK7, 13, 14, 18, and 19 (**a, c, e, g, i**). Central MEC showed immunoreactivity for CK7, 14, 18, and 19, but was negative for CK13 (**b, d, f, h, j**). Scale bars, 100 μm (**a–j**)

**Fig. 4:**
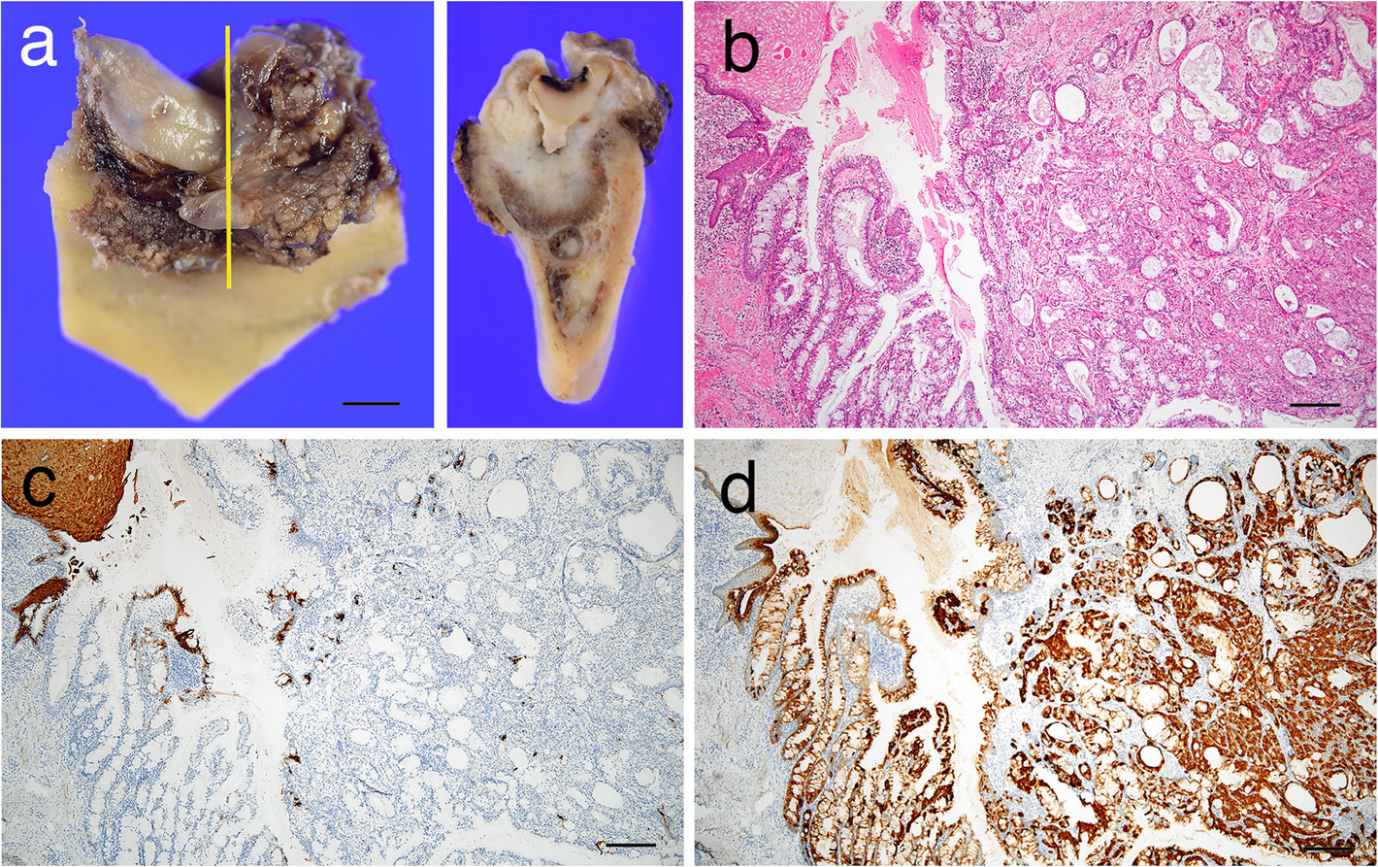
The surgical specimen after the biopsy showing central MEC. **a** The operative surgical specimen of the mandible. The specimen was cut before decalcification in a part (yellow line). The tumor expanded to the submucosal area from the mandibular bone in the cut surface (**b**) Tumor with many cystic spaces expanded into the submucosal area of the gingiva. **c, d **Immunoreactivity for CK18 was positive in central MEC, but negative for CK13. Scale bars, 10 mm (**a**), 200 μm (**b–d**)

We evaluated the cytokeratin (CK) profile by immunostaining to compare the CK expression patterns between central MEC and GOC in the biopsy specimen. The lining epithelium comprising non-keratinized squamous cells in the GOC part (Fig. 2f) showed immunopositivity for CK 7 (Fig. 3a), CK13 (Fig. 3c), CK14 (Fig. 3e), CK18 (Fig. 3g), and CK19 (Fig. 3i). On the other hand, the central MEC part was positively stained for CK7 (Fig. 3b), CK14 (Fig. 3f), CK18 (Fig. 3h), and CK19 (Fig. 3j), whereas immunoreactivity for CK13 was not detected (Fig. 3d). In the final surgical specimen obtained after mandibular partial resection, the tumor with several cystic spaces could be seen to expand into the submucosal area under the gingival mucosa from the mandibular bone in the cut surface (Fig. 4a). The resected specimen contained only central MEC (Fig. 4b). The keratin immunohistochemical profiles of CKs were similar to those of central MEC in the biopsy specimen, which was not positive for CK13 (Fig. 4c) but was positive for CK18 (Fig. 4d). The histopathological examination of the final surgical specimen confirmed the presence of central MEC arising from a previous GOC after consideration of the histopathological findings of the biopsy specimen.

**Fig. 5:**
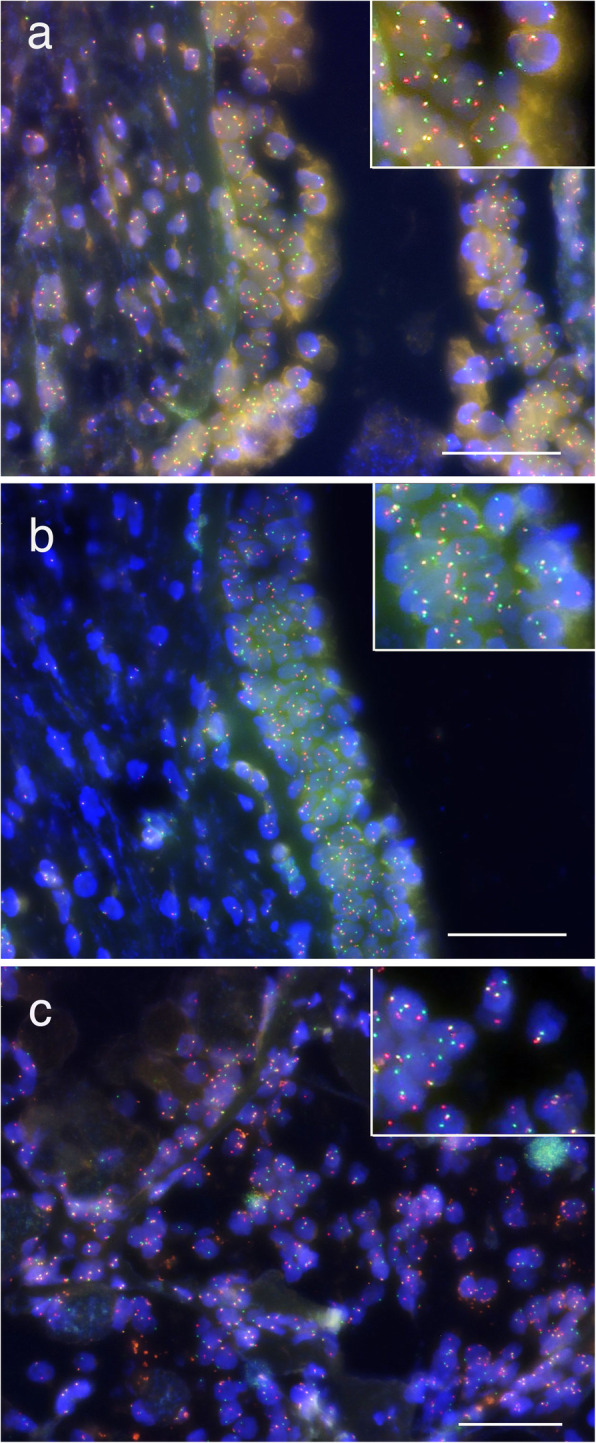
*MAML-*2 rearrangement by break-apart FISH in central MEC arising from a GOC. **a** The parts of central MEC in the biopsy specimen were positive for the *MAML-*2 rearrangement by break-apart FISH. **b** The *MAML-*2-split was also present in the parts of the GOC. **c** *MAML-*2 rearrangement was detected in the final surgical specimen of central MEC. Higher magnification images of FISH were inserted in **a** and **c**. Scale bars, 50 μm (**a–c**)

We sought to clarify the relationship of GOC to central MEC by performing *MAML*-2 molecular analysis of the lesion. Break-apart fluorescence in situ hybridization (FISH) for *MAML*-2 was performed. The component of central MEC in the biopsy specimen exhibited the *MAML*-2 rearrangement (Fig. 5a). In cystic areas of the GOC, the *MAML*-2-split was also present (Fig. 5b). Additionally, *MAML*-2 rearrangement was also detected in central MEC of the final surgical specimen (Fig. 5c).

## Discussion and conclusions

We described a rare case of central MEC arising from a GOC of the mandible. The GOC is an uncommon cyst accounting for 0.012 to 1.3 % of all cysts located in the facial part of the skull [4]. Central MEC is also very rare, representing only 2 to 4 % of all MECs [5]. Several cases formerly diagnosed as central MEC may have been cases of GOC and some central MECs could have originated from GOCs [4, 6]. There are previously reported cases in which the first biopsy was diagnosed as GOC, but the recurrent lesion was central MEC [4, 6]. To our knowledge, this is the first case report describing immunohistochemical and *MAML-2* rearrangement findings in a case of central MEC occurring directly from GOC. In other words, our case showed a cystic lesion with pathological findings typical of a GOC, while however also presenting a recognizable component of central MEC in the cyst wall.

Regarding the strong histopathological similarities between GOC and central MEC, previous reports have suggested that the differences in the expression pattern of CKs in GOC and central MEC may be helpful for diagnosis [5–8]. Our results demonstrated that while both central MEC and GOC expressed CKs 7, 14, 18, and 19, CK13 was exclusively expressed in GOC. Therefore, the immunohistochemical profile of CK13 may be useful for differential diagnosis of central MEC and GOC. Pires et al. compared the CK expression of GOC and central MEC and found differences in CK13 (100 % of GOC vs. 83 % of central MEC) [8]. Zhou et al. reported that 85.7 % of GOCs stained positive for CK13, whereas only 50 % of central MECs showed immunoreactivity for CK13 [5]. Our results were similar to those reported by Zhou et al. The GOCs were positive for CK13, whereas the central MECs were non-positive for CK13. Regarding CK13, we have also previously reported that the reciprocal immunohistochemical expression pattern of CK17 and CK13 in the oral mucosal epithelia corresponds to the grades of malignancy in oral squamous cell malignancies. Furthermore, we evaluated their immunohistochemical profiles by referring to the presence or absence of positivity as follows: the CK17+/CK13 − pattern indicated carcinoma in situ or squamous cell carcinoma, while the CK17−/CK13 + pattern meant normal and dysplastic epithelia [11, 12]. CK13 positivity can be a hallmark of squamous epithelium within the normal keratinization processes [11, 12].

Rearrangements of *MAML*-2 have recently been detected in up to 75 % of MECs of the salivary glands, and are very specific for this tumor type [3]. Bishop et al. reported *MAML*-2 rearrangements in central MECs; however, the *MAML*-2 status of GOCs is not known [3]. In our present case, *MAML*-2 rearrangements by break-apart FISH were present not only in the central MEC (both in the biopsy and final surgical specimen) but also in the cystic area of the GOC. Reddy et al. reported that rearrangements of *MAML*-2 are not always reliable for differentiating central MEC from GOC, as a lesion diagnosed as a cyst of unknown origin with features slightly suggestive of GOC was also positive for *MAML*-2 rearrangement [9]. In a study by Greer et al., *MAML*-2 rearrangements were detected in one case out of 11 previously diagnosed GOCs, and recurrent biologically aggressive GOCs with *MAML*-2 rearrangements were suggested to be a precursor of central MEC [10]. GOC is similar in histological features to central MEC and the *MAML*-2 rearrangements detected in GOC by break-apart FISH are the same as those observed in central MEC, suggesting that GOC may be of the same entity as central MEC.

Notably, odontogenic cysts are usually rather innocuous lesions that do not recur after curettage. Nevertheless, intraosseous carcinoma, including central MEC is associated with these cysts in 75 % cases [1]. Therefore, when a cystic lesion caused by an impacted tooth is extracted, the cyst wall needs to be properly removed surgically and it is important to request a histopathological examination to confirm the diagnosis.

## Abbreviations

MEC: Mucoepidermoid carcinoma
GOC: Glandular odontogenic cyst
MAML-2: Mastermind like − 2
FISH: Fluorescent in situ hybridization
CK: Cytokeratin
CT: Computed tomography
MRI: Magnetic resonance imaging
